# Psychometric testing of the caregiver contribution to self-care of coronary heart disease inventory

**DOI:** 10.1371/journal.pone.0302891

**Published:** 2024-05-10

**Authors:** Tatiana Bolgeo, Roberta Di Matteo, Niccolò Simonelli, Alberto Dal Molin, Barbara Bassola, Maura Lusignani, Antonio Maconi, Laura Rasero, Paolo Iovino, Ercole Vellone

**Affiliations:** 1 Research Training Innovation Infrastructure, Department of Research and Innovation, Azienda Ospedaliero-Universitaria SS Antonio e Biagio e Cesare Arrigo, Alessandria, Italy; 2 SC Cardiology, Azienda Ospedaliero-Universitaria SS Antonio e Biagio e Cesare Arrigo, Alessandria, Italy; 3 Department of Translational Medicine, University of Piemonte Orientale, Novara, Italy; 4 Health Professions’ Direction, Maggiore della Carità Hospital, Novara, Italy; 5 School of Nursing, University of Milan, Niguarda Hospital, Milan, Italy; 6 Department of Biomedical Sciences for Health, University of Milan, Milan, Italy; 7 Director of Department of Research and Innovation, Azienda Ospedaliero-Universitaria SS Antonio e Biagio e Cesare Arrigo, Alessandria, Italy; 8 Department of Health Sciences, University of Florence, Florence, Italy; 9 Department of Biomedicine and Prevention, University of Rome Tor Vergata, Rome, Italy; 10 Department of Nursing and Obstetrics, Wroclaw Medical University, Wroclaw, Poland; Universitat Ulm, GERMANY

## Abstract

**Background:**

Caregivers are important contributors to the self-care of patients with coronary heart disease (CHD).

**Aims:**

The aims of this study are to describe the development and psychometric properties of the caregiver contribution to self-care of coronary heart disease inventory (CC-SC-CHDI).

**Methods:**

The CC-SC-CHDI was developed from the patient version of the scale, the Self-care of Coronary Heart Disease Inventory (SC-CHDI) and translated into Italian using forward and backward translation. Baseline data from the HEARTS-IN-DYADS study were used. Confirmatory factor analysis (CFA) was conducted to assess factorial validity; Cronbach’s alpha and the model-based internal consistency index were used to test internal consistency reliability, and Pearson’s correlation coefficient was used to test convergent validity, by investigating the association between the CC-SC-CHDI and the SC-CHDI scores.

**Results:**

We included 131 caregivers (mean age 55 years, 81.2% females, 74% married) of patients affected by CHD (mean age 66 years, 80.2% males, 74% married). The CFA confirmed two factors in the caregiver contribution to self-care maintenance scale (“consulting behaviors” and “autonomous behaviors”), one factor for the CC to self-care monitoring scale, and two factors in the CC to self-care management scale (“consulting behaviors and problem-solving behaviors”). Reliability estimates were adequate for each scale (Cronbach’s alpha and model-based internal consistency indexes ranging from 0.73 to 0.90). Significant and positive correlations were observed between CC-SC-CHDI and SC-CHDI scales.

**Conclusion:**

The CC-SC-CHDI has satisfactory validity and reliability and can be used confidently in clinical settings and research to assess caregiver contributions to CHD self-care.

## Background

Coronary heart disease (CHD) represents the most common type of cardiovascular disease as well as the leading cause of morbidity and mortality worldwide [1]. A recent update on heart disease and stroke statistics indicates that approximately 244 million people are affected by CHD worldwide [2], and, despite a decreasing trend in mortality over the last few years, the burden of CHD remains high, with almost 4 million deaths in Europe [3].

Being diagnosed with CHD entails a starting process of lifetime adherence to self-care behaviors in order to prevent the onset of a new cardiac event and promote residual health [4]. Self-care has been defined as a process of health maintenance through health-promoting activities and disease management. This process is composed of three groups of behaviors: self-care maintenance, which includes all the behaviours performed to improve well-being, preserve health, and maintain physical and emotional stability in the course of illness (e.g. taking the prescribed medication, and performing physical activity); self-care monitoring, or the self-observance of symptoms or signs of the chronic illness a chronic disease (e.g. measuring blood pressure), and self-care management, which refers to any evaluation of signs and symptoms to decide whether and which specific actions are needed [5]. An adequate level of self-care is associated with favourable patient outcomes, including fewer readmissions [6], better survival [7], and improvement in quality of life [8].

Self-care in chronic illness frequently requires the support of they caregivers. Indeed, empirical research has shown that the presence of a caregiver is associated with better medication adherence, monitoring of symptoms, and physical activity involvement [9].

The caregiving process has been extensively studied in patients affected by heart failure, with investigations driven by the theory of caregiver contribution to self-care in heart failure [10]. This theory postulates that the informal caregiver, who is typically a family member or relative who cares for the patient at home, is critical for encouraging self-care; specifically, the caregiving behaviors can be supportive or substitutive in nature, depending on the patients’ autonomy, and are directed at sustaining all the spectrum of behaviors of self-care maintenance, monitoring, and management [10].

CHD is a disease with a chronic course that predisposes to an intense phase of caregiving process after discharge and during the acute phase of the illness. Despite this, the importance of caregiving support in CHD is remains an argument with a paucity of evidence. The existing literature reveals the high strain of the caregivers, especially during the most acute phase of the illness, which changed their role and their daily lives. Specifically, the study by Noonan et al., (2018) underlines that the caregivers generally did not feel prepared for the care of the family member after discharge, also due to the lack of knowledge of the illness and the trauma suffered during the cardiac event of their loved ones [11]. To the best of our knowledge, there has been no attempt to quantitatively categorize and examine the caregiving support in patients with CHD. This is not surprising, given that there is no instrument that quantifies the extent of caregiver contribution to self-care in CHD. Having a tool available to collect such information would be invaluable because quantifying caregiving support may guide strategies to optimize patient and caregiver in a collaborative approach to self-care, thereby minimizing adverse clinical outcomes, such as symptom exacerbations, and hospitalizations. Therefore, this article aims to describe the development and psychometric properties of the caregiver contribution to self-care of coronary heart disease inventory (CC-SC-CHDI).

## Methods

### Sampling and data collection

The baseline data from the HEARTS IN DYADS study were used for this psychometric analysis [12]. HEARTS IN DYADS was a longitudinal study aimed at describing patient self-care and caregiver contribution to self-care in CHD. Data were obtained from 5 Italian sites, representing the north and center part of Italy. After approval from the institutional review boards of Alessandria hospital (Northern Italy) (approval number 1244–06/08/2020). All potential participants received detailed explanations about the objectives, and methods of the study, and their degree of study commitment. The confidentiality and pseudo-anonymity of the data collected was also highlighted.” After obtaining the verbal and written informed consent, patients and caregivers were recruited at the inpatient hospital settings where their family members had been admitted for a coronary event onset. Enrolment was conducted during hospitalization, and specifically after the PTCA. Baseline questionnaires were administered to participants by face-to-face interviews. Subsequently, the dyads were contacted by telephone three and six months after recruitment. All the participants were at least 18 years old of age, able to read or write in Italian with cognitive integrity documented with a score less than 4 on the Six Item Screener [13]. In addition, patients with comorbidities for major cardiovascular conditions (e.g. congestive heart failure, cardiomyopathy) and unstable clinical conditions at the time of study enrolment (e.g. angina pectoris, dyspnea) were excluded. The caregivers included had to be the person inside or outside the family who provides most of the unpaid informal care to the patient. Recruitments began in May 2021 and ended in May 2023. First access to the baseline data of the HEARTS IN DYADS study occurred in December 2022.

### Instruments

The HEARTS-IN-DYADS study examined several aspects of self-care process: the predictors (i.e., patient and caregiver sociodemographic characteristics, severity of the CHD, comorbidities, caregiver preparedness, anxiety, depression, dual symptom management, and mutuality), the mediating role of patient and caregiver self-efficacy, and the outcomes of self-care (i.e., hospital admission, use of unplanned healthcare services, mortality). Specific tools were used to assess the predictors, process and outcomes of self-care and caregiver contribution to self-care. For this analysis we used caregiver data collected by the CC-SC-CHDI, the Self-care of Coronary Heart Disease Inventory Version 3.0 (SC-CHDI v.3) [14], the Caregiver Self-Efficacy in Contributing to patient Self-Care (CSE-CSC) [15], and a sociodemographic questionnaire.

The CC-SC-CHDI was developed by referring to the patient version of the scale (SC-CHDI), which was developed in English and also validated in the Italian language [14]. SC-CHDI consists of three scales with items formulated on 5-point Likert-type options: self-care maintenance (9 items), self-care monitoring (8 items) and self-care management (6 items). Each SC-CHDI scale has a standardized score with higher scores indicating better self-care.

The CC-SC-CHDI contains the same items and the same three scales as the SC-CHDI V3.0 patient version, except that the questions of each scale were worded to investigate how frequently the caregiver recommends self-care maintenance behaviors, monitoring behaviors, and how quickly he or she recognized his or her family member’s symptoms. For example, the introduction of the self-care maintenance SC-CHDI V3.0 scale asks the patient how often he/she performs a list of self-care behaviors; in the caregiver contribution version, it is asked the caregiver how often he/she recommends the patient the same behaviors of the SC-CHDI V3.0. This procedure has been used successfully to develop several other instruments measuring caregiver contribution to self-care in chronic conditions [16–19]. The CC-SC-CHDI was developed in English and Italian language but for this study, the Italian version was used. Also, the CC-SC-CHDI scales have a standardized 0–100 score with higher scores meaning better caregiver contribution to self-care. The data collected were checked for accuracy and precision, and were then manually entered into the REDCap® software [20].

The SC-CHDI v.3 is a 23-item instrument that measures the extent of self-care behaviors of patients with CHD. This instrument encompasses three scales that measure self-care maintenance (9 items), self-care monitoring (8 items), and self-care management (6 items) behaviors. Each scale has a standardized 0–100 score, with higher scores indicating better self-care. The SC-CHDI v.3 has shown satisfactory validity and reliability in a sample of Italian patients, with reliability estimates above 0.80 for all the three scales. For this study, we used the Italian version of the SC-CHDI v.3 to test the convergent validity of the CC-SC-CHDI.

The CSE-CSC is 10-item instrument that measures the extent of self-efficacy of the caregiver when contributing to patient self-care [15]. The score of this scale is standardized 0–100 with higher scores indicating higher self-efficacy. The CSE-CSC has showed adequate validity and reliability when tested on a sample with multiple chronic conditions (reliability indices between 0.90 and 0.97). The Italian version of the CSE-CSC was used for this study to test the convergent validity of the CC-SC-CHDI.

A sociodemographic questionnaire was developed ad hoc by the research team to collect the following variables: age, gender, marital status, occupation (e.g., retired), education, whether the patient lives with the caregiver, and the relationship the caregiver has with the patient (e.g., spouse or child).

### Sample size

Since this is a secondary analysis of the HEARTS-IN-DYADS study, we computed a post-hoc sample size estimation to verify the adequacy of the sample. At least ten individuals per item is suggested for confirmatory or exploratory factor analyses in order to ensure adequate inference of dimensionality and internal consistency [21]. Given that the CC-SC-CHDI was analyzed separately for each of the 3 scales (CC to maintenance, monitoring, and management), as previously done for similar caregiver contribution instruments [18, 19] and that the longest scale is composed of 9 items, a sample size of 90 patients was therefore deemed sufficient to address the primary study goal. However, we increased the size to 131 participants to account for possible dropouts and support a more stable analysis.

### Statistical analysis

The analysis was carried out in four steps. First, we investigated the sociodemographic characteristics of the sample of caregivers and their patients (such as gender and age), as well as the items of the CC-SC-CHDI scale (such as univariate and multivariate skewness and kurtosis); the description was presented with means, and standard deviations for continuous variables, and percentages and frequencies for categorical variables.

Second, we examined the CC-SC-SCHDI’s factorial validity; because this instrument is theory-based, we utilized confirmatory factor analysis to confirm the number of latent factors in the scale. This approach is consistent with other validation studies conducted on the measures of caregiving contribution [19, 22]. The three scales were tested separately, by maintaining the same factor structure of the SCHDI scale [14]. This decision was based on prior studies where the factorial structure of the caregiver contribution instruments reflected the same structure of the patients’ instruments [18, 22]. Aso, even though not specifically focused on CHD, the Middle-Range Theory of Self-Care of Chronic illness [5], the Situation-Specific Theory of Self-Care in Heart Failure [23] and the Situation-Specific Theory of Caregiver Contribution to Self-Care in Heart Failure [10] emphasize that patients and caregivers might have behaviors aimed at consulting healthcare providers (e.g., informing the healthcare provider about the symptom), behaviors that they can adopt in autonomy (e.g., do physical activity) or behaviors oriented to problem solving (e.g., take a medicine to make the symptom decrease or go away). For the CC to self-care maintenance scale, the following factors were hypothesized: consulting behaviors (items 1, 2 and 5) and autonomous behaviors (items 8, 6, 4, 3, 9, and 7). For the CC to self-care monitoring scale, we hypothesized one factor (items 10, 11, 12, 13, 14, 15 and 16). For the CC to self-care management scale, we hypothesized two factors: consulting behaviors (items 21 and 22), and problem-solving behaviors (items 18, 19, 20, and 23). The above factors have been identified also in prior analyses on caregiver contribution to self-care instruments [17, 18].

Comparative fit index (CFI), Tucker-Lewis Index (TLI), root mean square error of approximation (RMSEA), and standardized root mean square error of approximation (SRMR) were used to evaluate model fit [24]. CFI and TLI values of 0.90 or greater are indicative of supportive fit. RMSEA values of 0.08 or lower indicate a well-fitting model, and SRMR of 0.08 or lower indicate a satisfactory fit. Given its sensitivity to sample size, the χ^2^ statistic was also reported but not utilized to judge model fit.

Third, we tested the internal consistency reliability of each CC-SC-CHDI scale. We Cronbach’s alpha for unidimensional scales and model-based internal consistency index for multidimensional scale [25]. Both indexes should have a value of 0.70 or greater for satisfactory reliability. We also computed the item-total corrected correlations, which are adequate to discriminate the factor if the values are equal to or greater than 0.30 [26].

Fourth, we tested the construct validity of the CC-SC-CHDI. Specifically, we tested the hypothesis that the CC-SC-CHDI and the SCHDI were positively correlated, as also prior studies have demonstrated [19, 27]. We also tested the hypothesis that a higher self-efficacy in contributing to patient self-care should lead to higher CC to self-care, as described in the situation-specific theory of caregiver contribution by Vellone, Riegel [10].

SPSS® v.25 [28] was used for the descriptive and reliability analyses, while Mplus® v.8.6 [29] was used to perform the CFA. A p-value less than 0.05 was considered statistically significant.

## Results

### Description of the sample

Table 1 reports the characteristics of the participants. Most caregivers were the patient’s spouse (64.9%), had a mean age of 55 years (SD = 13.65) were female (80.2%), and lived with the patient (76.3%). Patients were 66 years old on average (SD = 11.20), mostly male (80.2%), and married (74%), with more than 40% having completed middle school.

**Table 1:** Sociodemographic characteristics of caregivers and patients with coronary heart disease.

	Patients (n = 131)	Caregivers (n = 131)
Characteristic	Mean (± SD) or n (%)	Mean (± SD) or n (%)
Age (years)	65.99 (11.20)	54.99 (13.65)
Gender (male)	105 (80.2)	26 (19.8)
Marital status (married)	97 (74)	97 (74)
Occupation (retired)	47 (35.9)	74 (56.5)
Education		
*No formal education*	17 (13)	6 (4.6)
*Elementary school*	43 (32.8)	33 (25.2)
*Middle school*	58 (44.3)	60 (45.8)
*High school*	11 (8.4)	27 (20.6)
*University degree*	2 (1.5)	5 (3.8)
Live with patient (yes)	-	100 (76.3)
Relationship with patient		
*Spouse/Partner*	-	85 (64.9)
*Child*	-	32 (24.4)
*Other*	-	14 (10.7)

**Legend.** CCI, Charlson Comorbidity Index; SD, standard deviation.

### Description of the items

The response rate of the CC-SC-CHDI was 100% and no missing data was reported. Table 2 reports the descriptive statistics of the items. Concerning the CC to self-care maintenance scale, the highest score mean was on item 1 (“keep appointments with the healthcare provider”), and the item with the lowest score was item 3 (“do something to relieve stress”). The highest score on the CC to self-care monitoring scale was on item 10 (“monitor your condition”), while the lowest score was found on item 17 (“how quickly did you recognize it as a heart symptom”). The CC to self-care management highest score was on the item 21 (“call the healthcare provider for guidance”), while the lowest score was on the item 19 (“take an aspirin”). Finally, the CC-SC-CHDI scales of self-care maintenance, monitoring, and management had scores which were lower than the recommended cut-off of 70 [30].

**Table 2:** Characteristics of the items of the caregiver contribution to self-care of coronary heart disease inventory (n = 131).

Items	Mean (SD)	Min	Max	Skewness	Kurtosis	Corrected item-total correlation
**Caregiver contribution to self-care maintenance scale**						
*How often do you recommend these things to the person you care for*? *(Or*, *how often do you do these activities because the person you care for is not able to do them)*						
Illness-related behaviors (3 items)						
1. Keep appointments with the healthcare provider	3.55 (1.50)	1	5	-0.59	-1.14	0.55
2. Take aspirin or other blood thinner	3.08 (1.62)	1	5	-0.14	-1.62	0.52
5. Take prescribed medicines without missing a dose	3.46 (1.62)	1	5	-0,.50	-1.40	0.67
Health-promoting behaviors (6 items)						
8. Eat fruits and vegetables	3.54 (1.48)	1	5	-0.46	-1.29	0.62
6. Ask for low fat items when eating out or visiting others?	2.95 (1.49)	1	5	0.08	-1.42	0.44
4. Do physical activity (e.g., take a brisk walk, use the stairs)	2.96 (1.43)	1	5	0.07	-1.34	0.49
3. Do something to relieve stress (e.g., medication, yoga, music)	2.47 (1.44)	1	5	0.35	-1.18	0.53
9. Avoid cigarettes and/or smokers	3.41 (1.68)	1	5	-0.46	-1.50	0.46
7. Try to avoid getting sick (e.g., flu shot, wash your hands)?	3.50 (1.52)	1	5	-0.50	-1.26	0.63
Total scale score (0–100)*	56.91 (25.27)	0	100	-0.12	-0.67	-
**Caregiver contribution to self-care monitoring scale**						
*Listed below are common things that people with coronary heart disease monitor*. *How often do you recommend these things*? *Or*, *do these things because the person you care for is not able to do them*?						
10. Monitor their condition?	3.16 (1.36)	1	5	-0.13	-1.22	0.72
11. Pay attention to changes in how they feel	3.14 (1.35)	1	5	-0.19	-1.17	0.72
12. Check the blood pressure	3.01 (1.40)	1	5	-0.06	-1.27	0.74
13. Monitor whether they tire more than usual doing normal activities	3.09 (1.32)	1	5	-0.21	-1.11	0.77
14. Monitor for medication side-effects	2.73 (1.45)	1	5	0.23	-1.30	0.75
15. Monitor for symptoms	3.13 (1.37)	1	5	-0.22	-1.14	0.76
16. Monitor body weight	2.88 (1.36)	1	5	-0.02	-1.30	0.55
*Many people with heart disease have symptoms of chest pain*, *chest pressure*, *burning*, *heaviness*, *shortness of breath*, *and fatigue*. *The last time the person you care for had a symptom …*						
17. How quickly did you recognize it as a heart symptom?	2.14 (1.77)	0	5	0.24	-1.31	-
Total scale score (0–100)*	51.25 (27.72)	0	100	-0.11	-0.97	-
**Caregiver contribution to self-care management scale**						
*Listed below are behaviors that people with heart disease use to control their symptoms*. *When the person you care for has symptoms*, *how likely are you to recommend that they use one of these*? *Or*, *do these because the person you care for is not able to do them*?						
Consulting behaviors (2 items)						
21. Call the healthcare provider for guidance	3.90 (1.37)	1	5	-1.02	-0.28	0.58
22. Tell the healthcare provider about the symptom at the next office visit	3.88 (1.34)	1	5	-0.91	-0.45	0.61
Problem-solving behaviors (4 items)						
18. Change the activity level (slow down, rest)	3.47 (1.22)	1	5	-0.24	-0.93	0.50
19. Take an aspirin	2.71 (1.50)	1	5	0.24	-1.37	0.54
20. Take a medicine to make the symptom decrease or go away	3.04 (1.49)	1	5	-0.95	-1.38	0.59
*Think of what you did the last time the person you care for had a symptom of heart disease*.						
23. Did the treatment they used make them feel better?	2.57 (1.77)	1	5	-0.05	-1.28	0.40
Total scale score (0–100)*	59.72 (23.97)	0	100	-0.29	-0.43	-

**Legend.** SD, standard deviation.

*Raw score range.

Skewness and kurtosis values did not exceed ±1 and ±3, respectively, suggesting univariate normality. Mardia χ^2^ test was significant both for skewness and kurtosis (p < 0.001), indicating violation of multivariate normality. In order to compensate for this departure, we performed the CFA models using the robust maximum likelihood estimation (MLR).”

### Factorial validity testing

#### CC to self-care maintenance scale

**Fig 1:**
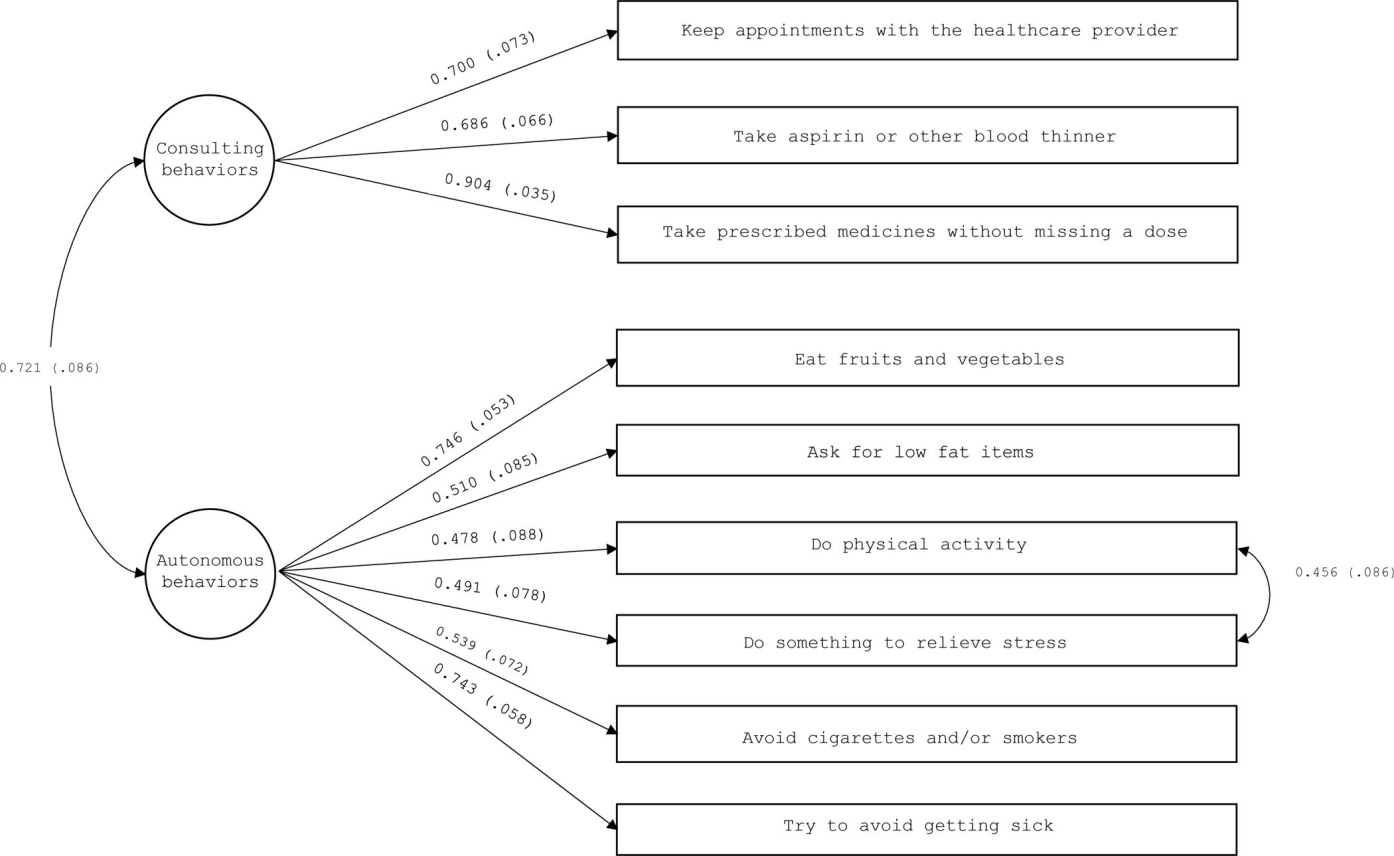
Confirmatory factor analysis of the caregiver contribution to self-care maintenance scale (n = 131). Rectangles represent observed variables; circles represent latent factors. Numbers near the 1-headed arrows are factor loading coefficients; numbers near the 2-headed arrows are correlation coefficients. Loadings are completely standardized and all statistically significant.

CC to self-care maintenance scale was specified as encompassing the two factors of “illness related behaviors” (items 1,2, and 5), and “health promoting behaviors” (items 3,4, and 6–9). The fit indices of this model were unsatisfactory: χ2 (26, N = 131) = 60.02, P = < 0.001, CFI = 0.88, TLI = 0.83, RMSEA = 0.099 (90% CI, 0.066–0.132), P = 0.009, and SRMR = 0.074. Inspection of the modification indices showed an excessive covariance between items 4 (“do physical activity”) and 3 (“do something to relieve stress”). Specification of this covariance is theoretically justified, given that sometimes people use physical activity to relieve stress. Moreover, according to Bagozzi and Fornell (1983), this analytical approach is reasonable as far as the covariances are theoretically plausible and their specification does not alter the estimates of the other model parameters, such as in our case. After rerunning the model with this covariance, the fit improved considerably: χ2 (25, N = 131) = 39.92, P = 0.03, CFI = 0.95, TLI = 0.92, RMSEA = 0.067 (90% CI, 0.021–0.104), P = .221, and SRMR = 0.061. The factor loadings were all significant and high. The two factors were significantly correlated at 0.72 (p < .001) (Fig 1).

#### CC to self-care monitoring scale

**Fig 2:**
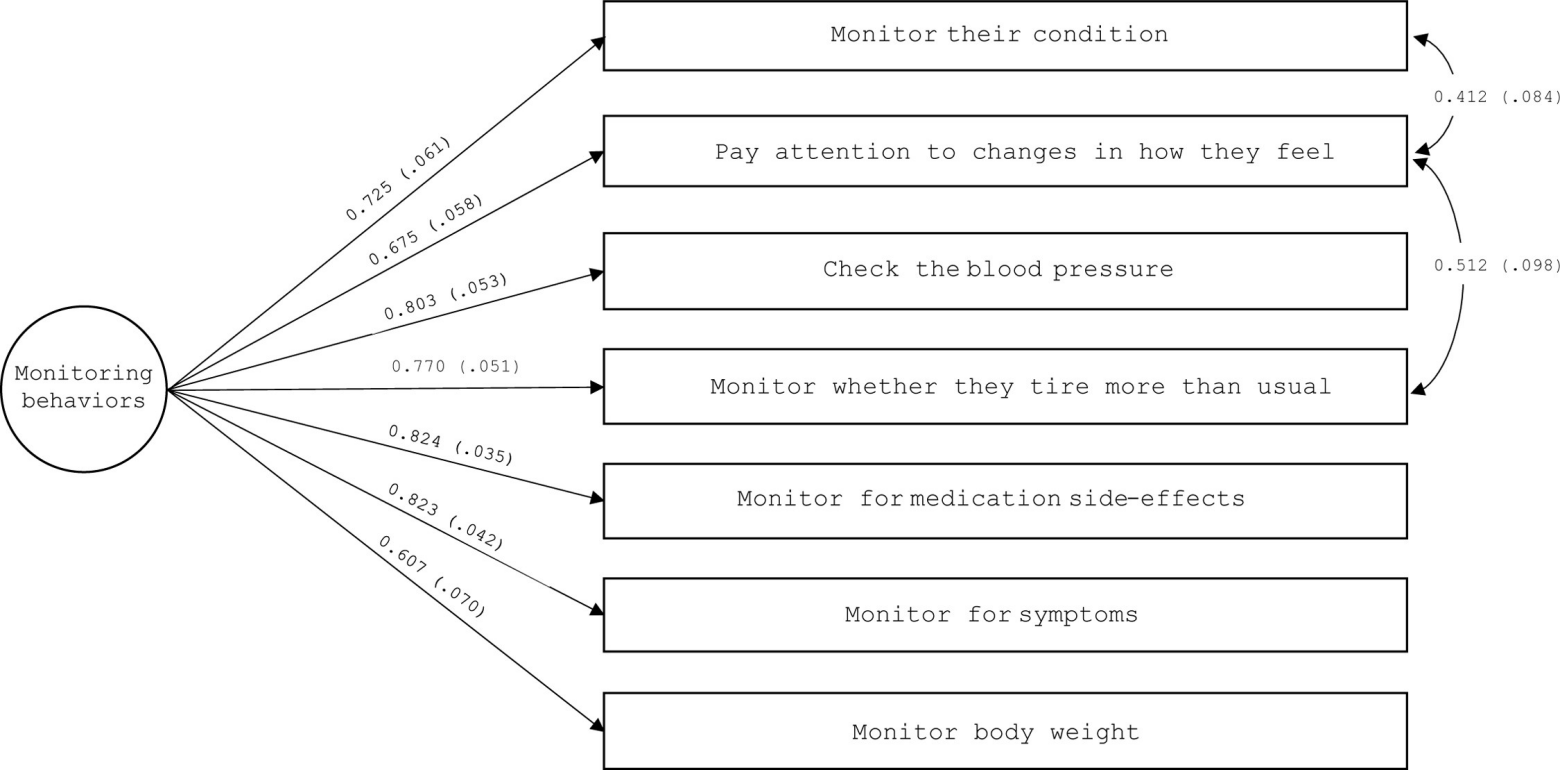
Confirmatory factor analysis of the caregiver contribution to self-care monitoring scale (n = 131). Rectangles represent observed variables; circles represent latent factors. Numbers near the 1-headed arrows are factor loading coefficients; numbers near the 2-headed arrows are correlation coefficients. Loadings are completely standardized and all statistically significant.

We theorized that a single factor would explain the correlations between the seven items of the scale; therefore, the CFA was specified as a single factor, whose fit indices were unsatisfactory: χ2 (14, N = 131) = 37.74, P = <0.001, CFI = 0.92, TLI = 0.88, RMSEA = 0.113 (90% CI, 0.070–0.157), P = 0.010, and SRMR = 0.047. Modification indices suggested estimating two error covariances. The first was between items 10 (“monitor their condition”) and 11 (“pay attention to changes in how you feel”), and the second was between items 11 and 13 (“monitor whether they tire more than usual doing normal activities”). These covariances are explained by that they all address the function of body listening [5]. Given that their specification was theoretically justified and did not change parameter estimates, the model was respecified with these covariances, whereupon the fit was excellent: χ2 (12, N = 131) = 8.07, P = 0.780, CFI = 1.00, TLI = 1.00, RMSEA < 0.001 (90% CI, 0.00–0.06), P = 0.920, and SRMR = 0.02. All factor loadings were high and significant (Fig 2).

#### CC to self-care management scale

**Fig 3:**
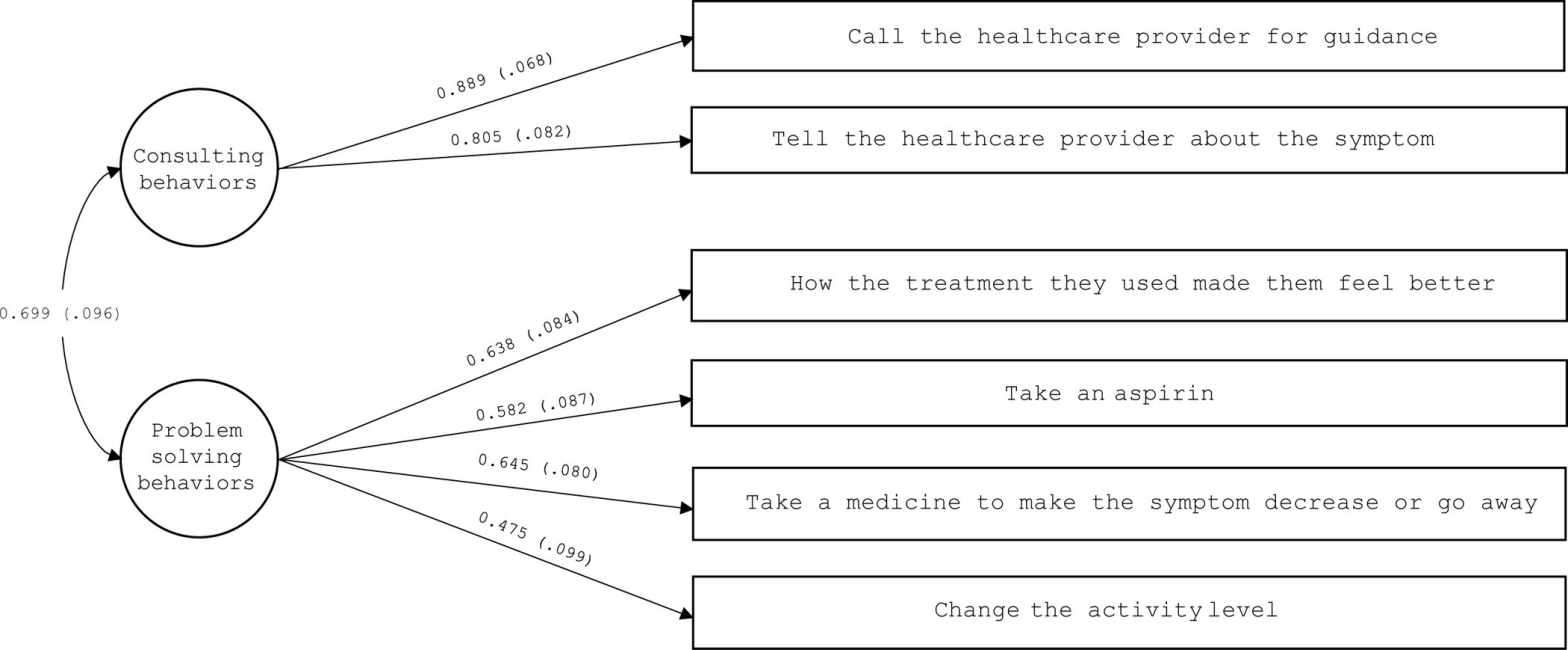
Confirmatory factor analysis of the caregiver contribution to self-care management scale (n = 131). Rectangles represent observed variables; circles represent latent factors. Numbers near the 1-headed arrows are factor loading coefficients; numbers near the 2-headed arrows are correlation coefficients. Loadings are completely standardized and all statistically significant.

We theorized that two factors would explain the correlations between the six items of the scale; therefore, the CFA was specified as a two-factor model, which yielded satisfactory fit indices: χ2 (8, N = 131) = 13.94, P = 0.083, CFI = 0.96, TLI = 0.93, RMSEA = 0.075 (90% CI, 0.00–0.139), P = 0.232, and SRMR = 0.04. All factor loadings were significant and adequate (Fig 3).

### Internal consistency reliability testing

Internal consistency reliability of the CC to self-care maintenance scale was measured with the model-based internal consistency index as this scale resulted multidimensional at CFA. Reliability was adequate at 0.79 and all the items had item-total corrected correlations greater than 0.40.

Internal consistency reliability of the CC to self-care monitoring scale was computed with Cronbach’s alpha as this scale resulted unidimensional at CFA. Cronbach’s alpha was 0.90. All the item-total corrected correlations were greater than 0.50.

Internal consistency reliability of the CC to self-care management scale was measured with the model-based internal consistency index as this scale was multidimensional and resulted with a coefficient of 0.73. Factor determinacy scores for the consulting and problem-solving behaviors factors were adequate at 0.90 and 0.85, respectively. All the items had item-total corrected correlations greater than 0.40.

### Convergent validity testing

Table 3 shows the Pearson correlation coefficients computed to examine the relationship between the constructs of interest. When the CC-SC-CHDI scores were analyzed in relation to the SC-CHDI V3.0 scores, we found significant positive correlations ranging from 0.23 to 0.42 (p = 0.01). When we examined the relationship between the CC-SC-CHDI and the self-efficacy in contributing to patient self-care, we found significant positive correlations ranging from 0.25 to 0.41 (p = 0.01).

**Table 3:** Correlations between the dimensions of self-care of coronary heart disease inventory and those of the caregiver contribution to self-care of coronary heart disease inventory.

Variables	CC to self-care maintenance	CC to self-care monitoring	CC to self-care management	Self-efficacy in CC to patient self-care	Self-care maintenance	Self-care monitoring	Self-care management
CC to self-care maintenance	1						
CC to self-care monitoring	0.58**	1					
CC to self-care management	0.51**	0.56**	1				
Self-efficacy in CC to patient self-care	0.29**	0.32**	0.49**	1			
Self-care maintenance	0.41**	0.37**	0.25**		1		
Self-care monitoring	0.31**	0.42**	0.23**			1	
Self-care management	0.30**	0.23**	0.35**				1

**Note**. CC, Caregiver Contributions.

** significant at p = 0.01.

## Discussion

The results of this study suggest that the CC-SC-CHDI has satisfactory validity and reliability properties; thus, it can initially be used in clinical practice to measure the caregiving contributions to the self-care of patients affected by CHD. To the best of our knowledge, this is the first study to introduce and examine the psychometric properties of such an instrument in a sample of caregivers of CHD patients. Previous research has underscored the importance of the caregivers’ role in the care of these patients [31, 32]; consequently, having a valid and reliable instrument available to quantify this support is critical.

The CC-SC-CHDI makes important contributions to the development of the self-care sciences in chronic illnesses. First and foremost, the CC-SC-CHDI is theory-based, because it was derived from the theory of self-care of chronic illness [5]. Second, the fact that the factorial structure of this scale is similar to that of the SC-CHDI V3.0 indicates that, also in the context of CHD, patients and caregivers share the same construct of the self-care process.

We found two dimensions within the CC to self-care maintenance scale: consulting behaviors and autonomous behaviors. Interestingly, the excessive covariance we found between item 4 “do physical activity” and item 5 “do something to relieve stress”, suggests that caregivers elicit physical activity in patients in order to cope with their stress. This hypothesis is reinforced by the results we found in the patient version of the scale, where the same covariance emerged [14]. The high correlation emerged between the factors of consulting and autonomous behaviors provides further proof that these latent factors faithfully represent the CC to self-care maintenance behaviors.

We found that the CC to self-care monitoring scale was unidimensional. The specified covariances emerging from the modification indices suggest that fatigue may be one of the predominant symptoms under vigilance by the caregivers (due to error correlations between the items asking whether they monitor patient’s tiredness and feelings). In CHD patients, the prevalence of fatigue has been found to range from 39% during cardiac rehabilitation, to 28% one year after diagnosis [33]. Most importantly, fatigue has been found to be multidimensional, with components of mental fatigue and reduced activity being more prominent than others [34]. This could help explain why caregivers seem to focus on fatigue when helping patients monitor their condition and pay attention to changes in their feelings.

Regarding the CC to self-care management scale, we confirmed two dimensions: “consulting behaviors” and “problem solving behaviors”. The same structure was also found in the SC-CHDI [14], suggesting that both patients and caregivers have similar conceptions about the nature of the actions they perform during the management of the disease. This is important, because, as shown by other studies, it is more likely that the dyad will achieve a higher level of congruence in this scenario, and have better patient outcomes, than when both members are discordant in their decisions [35, 36].

The CC-SC-CHDI showed adequate internal reliability in our study, which is comparable with psychometric analyses carried out on previous self-care scales [16, 22]. Moreover, despite the discovery of a 2-factor structure for the CC to self-care maintenance and management scales, the multidimensional reliability index was adequate, supporting the use of a unique score for the factor.

Finally, the medium-to-large correlations between the CC-SC-CHDI and SC-CHDIV3.0 and self-efficacy confirmed convergent validity. This was expected because CHD patient self-care and CC to CHD self-care are theoretically and empirically interrelated [10], and self-efficacy is known to be a powerful predictor of all CC to self-care behaviors.

This study suffers a few limitations that are worth mentioning. First, the convenience sampling adopted by our study may have led to selection bias. For example, we intentionally excluded patients with average-to-severe cognitive impairment; therefore, it is not clear whether inclusion of these patients would have led to different findings. Generalization of the findings to other cultures is not advisable, since the effects of diverse healthcare system organizations, and the variability in socioeconomic conditions were identified as important obstacles of the self-care process. Second, we did not investigate the stability of the instrument over time; thus, future studies should implement test-retest analyses to confirm that the responses to the items and the scores are temporally stable. Finally, we need to acknowledge that the CC-SC-CHDI is a self-report measure, which, like other instruments of this kind is subjected to method bias. One major bias is specifically social desirability, which might have impacted the validity and reliability of our instrument.

## Conclusions

This study demonstrates that the CC-SC-CHDI is a psychometrically sound instrument with satisfactory validity and reliability. Consequently, we recommend it to be initially used both in clinical and research settings to measure CC to CHD self-care. Future studies should be conducted to test this instrument in other cultures and populations with different social levels, which potentially detect differences in caregiving support; this would help enrich the understanding of the CC experience in CHD populations.

## Supporting information

S1 Dataset (XLSX)
